# Progression-Free Survival versus Overall Survival in Patients with PSA-Recurrent Prostate Cancer: Long-Term Analysis of a Randomized Phase 2 Trial of pTVG-HP versus Placebo

**DOI:** 10.21203/rs.3.rs-8843559/v1

**Published:** 2026-02-22

**Authors:** Douglas McNeel, Jens Eickhoff, Arianna Perez, Glenn Liu

**Affiliations:** University of Wisconsin Comprehensive Cancer Center; University of Wisconsin-Madison; University of Wisconsin-Madison

**Keywords:** DNA vaccine, prostate cancer, prostatic acid phosphatase, clinical trial, overall survival

## Abstract

**Background:**

We previously reported results from a randomized phase 2 trial evaluating a DNA vaccine (pTVG-HP) versus placebo (GM-CSF vaccine adjuvant alone) in patients with PSA-recurrent, nonmetastatic, non-castrate prostate cancer (NCT01341652). The primary endpoint of 2-year metastasis-free survival (MFS) was not different between the treatment arms. This trial was reopened for long-term evaluation at one study site.

**Methods and Results:**

The median follow-up for long-term overall survival was 4.1 years (range 0.1–13.8+ years). Median overall survival of 48 patients treated with pTVG-HP was 13.4 years versus 8.6 years for 49 patients treated with placebo (log-rank test p-value=0.020). Long-term follow-up information on survival and subsequent treatments was available for 59 patients. Of these, 56 were treated with subsequent androgen deprivation therapy (ADT). When analyzing overall survival for this cohort, the adjusted hazard ratio was 0.56 (p=0.021) when comparing pTVG-HP vs. placebo, after adjusting for ADT and subsequent treatment exposure. The median time to beginning ADT was 2.2 years for patients randomized to pTVG-HP versus 1.4 years for patients randomized to placebo (p=0.192). The median time to the next therapy for castration-resistant disease after beginning ADT was 4.7 years for patients randomized to pTVG-HP versus 2.1 years for patients randomized to placebo (p=0.112).

**Conclusions:**

These results suggest that pTVG-HP may have had single-agent benefit that could not be appreciated using a MFS endpoint, challenging the notion that MFS can uniformly serve as a surrogate endpoint for overall survival in this stage of disease.

## INTRODUCTION

Nearly a third of patients with prostate cancer have recurrence after primary treatment for localized disease.^1^ Recurrence is typically heralded by an increase in serum PSA, which then prompts radiographic restaging. This recurrent stage of prostate cancer represents a heterogeneous population, as many patients can do well for many years before developing overt metastatic disease.^2^ Patients with rapidly rising PSA (shorter PSA doubling time) are at highest risk for metastatic progression and death from prostate cancer.^3^ Prior to 2020, most patients with this stage of disease were treated with androgen deprivation therapy (ADT), or observed until they had evidence of metastatic disease by conventional imaging (CT scans and bone scans), at which point ADT was initiated. With more widespread implementation of PSMA PET/CT imaging, and identification of metastases not visible by conventional imaging, many patients are now treated with targeted radiation therapy, which can delay the progression of disease.^4^ Single agent enzalutamide was approved in 2024 for this stage of disease, based on the demonstration that it prolonged the time to the development of metastases.^5^ While enzalutamide monotherapy did not lead to improved overall survival compared to standard ADT, the combination of enzalutamide and ADT did demonstrate improved overall survival compared to ADT monotherapy in patients with rapid (< 9 months) PSA doubling time and has recently become a standard approach to the treatment of patients with this stage of disease.^6^ Notwithstanding, may patients with biochemically recurrent prostate cancer still forgo these treatments to avoid the effects of androgen deprivation.

We previously reported the results of a multi-institutional randomized phase 2 trial (NCT01341652), conducted between 2011 and 2016, in patients with PSA-recurrent prostate cancer without evidence of metastases by conventional imaging.^7^ All patients had a pre-treatment PSA doubling time less than 12 months, effectively selecting individuals at greatest risk for metastatic disease progression. Patients were randomized to receive a DNA vaccine encoding prostatic acid phosphatase (PAP, pTVG-HP) with GM-CSF co-administered as a vaccine adjuvant, or GM-CSF alone without vaccine. The primary endpoint was 2-year metastasis-free survival (MFS). The trial was negative for that primary endpoint, although a planned subset analysis did observe a longer MFS in patients with the most aggressive disease (pre-treatment PSA doubling time less than 3 months).^7^ A small (n = 22) phase 1 trial with pTVG-HP had previously been conducted in the same population of patients with biochemically-recurrent prostate cancer, although irrespective of pre-treatment PSA doubling time.^8^ Because DNA vaccines were relatively new when that trial was conducted between 2005 and 2007, FDA had requested 15 years of follow up. We recently reported the 15-year follow-up of that small trial, in which the median overall survival was 12.3 years, and 23% were alive at 15 years.^9^ Patients who subsequently began ADT were on treatment a median of 6.4 years before beginning another therapy. In addition, we observed that 60% (6/10) patients had vaccine antigen-specific T cells detectable many years after treatment.^9^ Given these findings, we reopened the randomized phase 2 trial (NCT01341652) for longer term analysis, to determine whether treatment might have impacted overall survival.

## MATERIALS AND METHODS

Clinical Trial and Regulatory Information: A randomized phase 2 trial of a DNA vaccine encoding prostatic acid phosphatase (pTVG-HP, aka MVI-816) in patients with progressive, nonmetastatic, castration-sensitive prostate cancer (NCT01341652) was conducted between 2011 and 2016 at three academic medical centers (University of Wisconsin Carbone Cancer Center, University of California San Francisco, and Johns Hopkins University). The study protocol had been reviewed and approved by all local and federal (FDA, NIH Recombinant DNA Advisory Committee) entities, and all patients gave written IRB-approved consent for participation. In total, 99 patients were enrolled in this trial with 49 patients randomized to placebo (200 μg GM-CSF adjuvant given intradermally) and 50 to vaccination (100 μg pTVG-HP with 200 μg GM-CSF adjuvant, given together intradermally). Patients were required to have a pre-treatment PSA doubling time (DT) of less than 12 months, and were stratified based on pre-treatment PSA DT (0–3 months, 3–6 months, or 6–12 months), initial Gleason score (≤ 7 or > 7), and baseline PSA at the time of screening (≤ 10 ng/mL or > 10 ng/mL). Treatment was administered every 2 weeks over a 3-month period, and then every 3 months up to 2 years total. Conventional imaging (CT scans and bone scans) was performed at 6-month intervals or as clinically indicated. The primary objective was 2-year metastasis-free survival. Secondary objectives included safety, changes in PSA doubling time, and median progression-free survival. As part of the original protocol, patients were followed for an additional 2 years following their last treatment to obtain information including subsequent stage and treatment for prostate cancer, other new major medical problems, and date of death if patient was deceased. The protocol was reopened in 2025 at the University of Wisconsin lead site to collect these same elements in longer-term follow up of 59 patients treated at that site, and to specifically evaluate overall survival. It was not possible to reopen the trial at the other clinical trial sites.

Statistical Analysis: Overall survival (OS) was defined as the time period from the randomization date to the date of death from any cause (event) or last known survival status date (censored). OS was analyzed using the Kapan-Meier method and compared between groups using a log-rank test. Survival analyses were conducted on two cohorts (1) all eligible randomized study participants, i.e., study participants with and without long-term follow-up survival assessments, (2) all eligible randomized study participants for whom long-term follow-up survival and subsequent treatment assessments were available. Summary statistics of the baseline characteristics were presented for the two cohorts. For the cohort of patients with long-term survival and subsequent treatment assessments, the comparison of OS between study arms was also conducted using a Cox regression model, where ADT and any subsequent therapy exposure were included as time-varying covariates. The adjusted hazard ratio (aHR) was reported, along with the corresponding 95% confidence interval (CI). For patients treated with ADT, time from randomization to the initiation of ADT was analyzed using the Kaplan-Meier method and compared between study arms using the unstratified log-rank test. Analogously, for patients treated with subsequent therapy for castration-resistant disease following ADT, the time from the start of ADT to the initiation of any subsequent therapy for recurrent prostate cancer was analyzed using the Kaplan-Meier method and compared between study arms using the unstratified log-rank test. All reported P-values are two-sided and P < 0.05 was used to define statistical significance. Statistical analyses were conducted using SAS software (SAS Institute Inc., Cary NC), version 9.4.

## RESULTS

**Figure 1:**
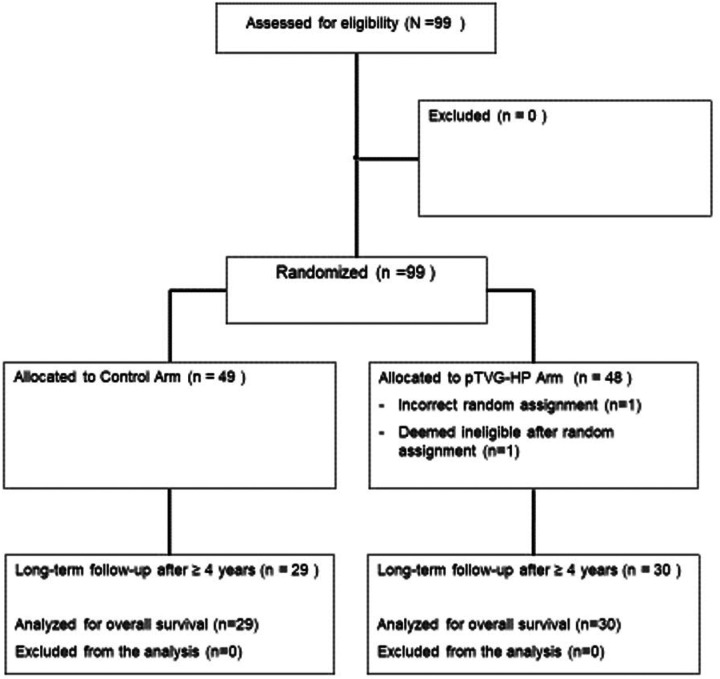
Patient allocation: Shown is the CONSORT diagram for patients previously treated on the NCT01341652 trial for the current analysis.

99 patients with biochemically recurrent prostate cancer, without evidence of metastases by conventional imaging (CT scans and bone scintigraphy), were treated in a clinical trial (NCT01341652) in which they were randomized 1:1 to receive 100 μg pTVG-HP, a plasmid DNA encoding PAP, delivered intradermally with GM-CSF co-administered as a vaccine adjuvant, or GM-CSF alone. The primary endpoint was 2-year MFS. Secondary clinical endpoints included median MFS, and changes in PSA DT. Patients were followed for 2 years following completion of the trial for safety analysis and to identify subsequent treatments. Given this early stage of disease, overall survival was not anticipated as a primary or secondary endpoint. The results from that trial were previously reported in 2019, demonstrating no difference between study arms (2-year MFS 41.8% versus 42.3%, p = 0.97).^8^ The trial was reopened in 2025 at the lead site (University of Wisconsin, minimal risk IRB approval) to collect longer term data as in the original protocol (including subsequent treatments, and date and cause of death, or last known date alive). Data for participants at the other clinical trial sites beyond the initial 2 years of post-treatment follow-up could not be obtained. The allocation of individuals for analysis is indicated in the CONSORT diagram in Fig. 1, and their baseline characteristics relative to the entire treatment group are shown in Table 1.

**Figure 2:**
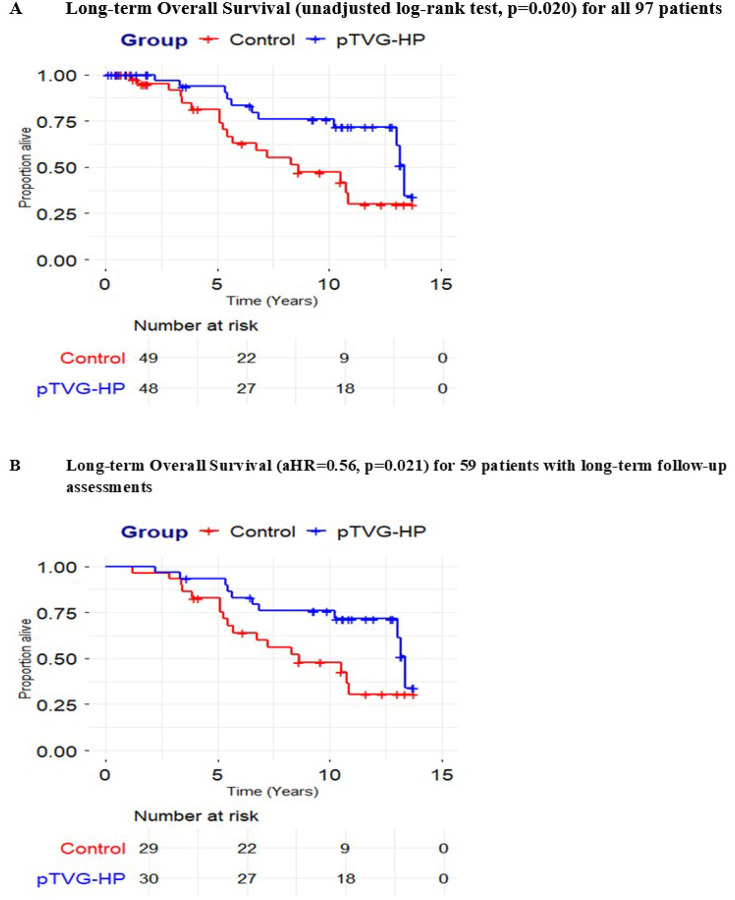
Clinical endpoints: Panel A: Kaplan-Meyer plot of overall survival, defined as the time from original trial randomization to the date of death or censored at the last known date alive for all 97 patients. Panel B: Kaplan-Meyer plot of overall survival for 59 patients with available long-term data for survival and subsequent treatments. Comparison of overall survival between groups was conducted using a Cox regression model with time varying covariates (ADT and subsequent therapy exposure) and reported as aHR.

**Figure 3:**
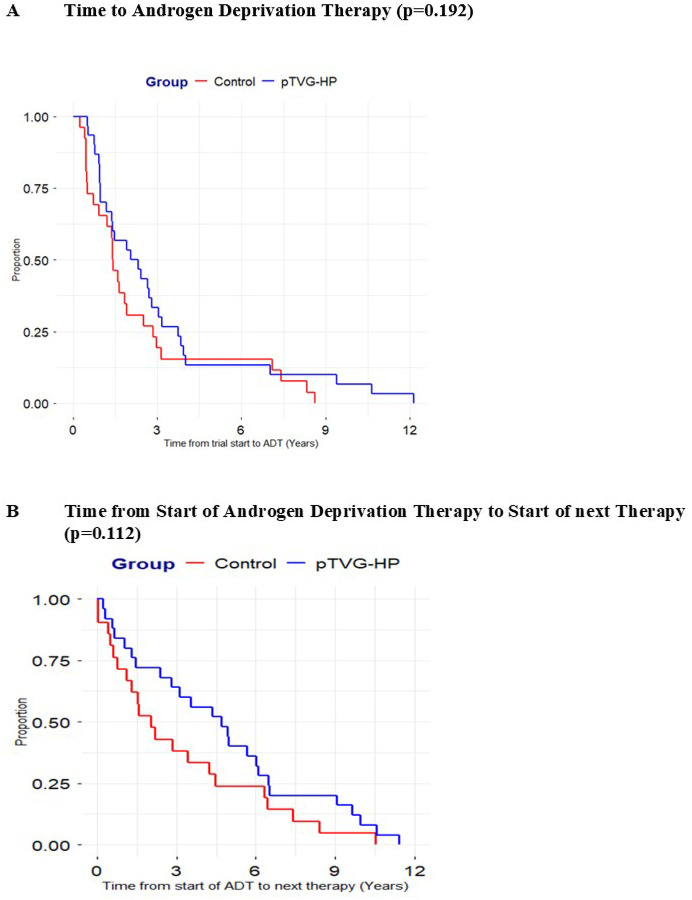
Duration of ADT and subsequent treatment exposure: Panel A: Kaplan-Meyer plots of time to beginning ADT from the original trial randomization date for the two treatment arms. Comparison between treatment arms was conducted using a log-rank test. Panel B: Kaplan-Meyer plots of time on ADT, from date of initiation of ADT to date of initiating next therapy for progressive disease, for the two treatment arms Comparison between treatment arms was conducted using a log-rank test.

As shown in Fig. 2A, when analyzing patients with and without long-term follow up survival assessment (n = 97), patients randomized to the pTVG-HP arm had a longer median overall survival than patients randomized to the placebo arm (13.4 years versus 8.6 years, unadjusted log-rank test p-value = 0.021). Similar results were observed when analyzing only the 59 patients with long-term follow-up survival assessments (Fig. 2B). The median overall survival for patients randomized to the placebo arm was 8.6 years vs. 13.4 years randomized to the pTVG-HP arm (unadjusted log-rank test p-value = 0.032, aHR = 0.56, 95% CI: 0.34–0.92, p-value = 0.021). The time to beginning ADT (LHRH agonist/antagonist +/− bicalutamide) in 56 patients treated with ADT was not different between treatment arms (median 2.2 years versus 1.4 years, HR = 0.70, p = 0.192, Fig. 3A). This was not unexpected, given that the trial was negative for a difference in the time to metastatic disease, and patients at our center typically began ADT at the time metastatic disease was identified by conventional imaging. Furthermore, the median time to initiation of the next systemic therapy after ADT in 46 patients treated with a subsequent therapy for castration-resistant disease was 4.7 years in patients randomized to the pTVG-HP arm versus an expected 2.1 years in patients randomized to the placebo arm (HR = 0.62, p = 0.116, Fig. 3B). Supplemental Table 1 shows the subsequent therapies for castration-resistant disease received by each individual.

## DISCUSSION

Patients with rising serum PSA following definitive treatment for localized prostate cancer are at increased risk of developing radiographic recurrence and death from prostate cancer. Even in a contemporary cohort of 781 patients, many of whom were treated with systemic therapy prior to the development of overt metastases detectable by conventional scans, the median overall survival was less than 12 years.^10^ Despite this, the development and evaluation of treatments for patients with PSA-recurrent disease has been challenging due to the heterogeneity of this stage of disease and its long natural history.^2^ Patients with rapidly rising PSA, typically measured by PSA doubling time, are at highest risk for early recurrence, and it has been suggested that this may be the population most in need of therapy.^11^ Enzalutamide monotherapy has been approved for use in patients with PSA recurrent disease, and metastasis-directed radiation therapy is often used to treat lesions identified by PSMA PET/CT imaging in order to delay time to ADT. In the recent EMBARK trial in patients with a PSA DT less than 9 months, while enzalutamide monotherapy did not prolong overall survival relative to ADT alone, the combination of enzalutamide with ADT demonstrated an improvement in overall survival compared to ADT monotherapy (78.9% versus 69.5% survival at 8 years).^6^ This has now become a standard of care treatment for this population. However, despite the fact that enzalutamide was not demonstrated to reduce quality of life in this setting,^12^ many patients with biochemically recurrent prostate cancer are keen to avoid the use of ADT and/or enzalutamide, due to potential side effects and cost.

Given the long natural history and the challenge of using survival as a primary endpoint for clinical trials, it has been suggested that MFS should be used as a surrogate for overall survival in this stage of disease.^13^ In fact, a large meta-analysis performed by the international Intermediate Clinical Endpoints in Cancer of the Prostate (ICECAP) working group of 75 clinical trials found that of different clinical endpoints, MFS most strongly correlated with overall survival in patients with localized or biochemically recurrent prostate cancer.^14^ This concept is further supported by the results of the EMBARK trial. We had previously conducted the largest randomized phase 2 trial in this disease stage using an anti-tumor vaccine, for which the primary endpoint had been MFS. We now present a longer follow up of that trial in which patients treated with vaccine experienced a significantly longer overall survival, despite not demonstrating an overall improvement in MFS. These findings challenge the notion that MFS can uniformly serve as a surrogate for overall survival in this disease setting. Of note, we did previously observe an increase in MFS in patients with very rapid PSA doubling time, < 3 months, and thus those individuals with the shortest expected MFS, but not for the entire population.

Sipuleucel-T was approved in 2010 as a treatment for metastatic, castration-resistant prostate cancer based on improved overall survival observed in a randomized clinical trial, with data from other trials similarly supporting improved overall survival compared to placebo treatment.^15,16^ As such, it was the first anti-tumor vaccine approved by FDA as a treatment for existing cancer. Its approval was met with a lot of controversy, in part because previous trials had not demonstrated an impact on time to progression in trials using PFS as an endpoint, and because of the lack of PSA or objective responses observed.^17^ Modeling by other investigators suggested that cancer vaccines might slow the growth of cancer, potentially explaining this paradox of why time to progression might be less impacted than overall survival.^18^ If true, then this suggests that use of vaccines in earlier stages of disease should be even more beneficial. In fact, patients with lower volume of prostate cancer were found to have a greater survival benefit following treatment with sipuleucel-T in late stage prostate cancer.^19^ Long term data from the randomized phase 2 trial presented in this current report are further supportive of this concept. The substantially longer 5-year difference in overall survival observed between treatment arms is certainly clinically meaningful, and suggests that use of vaccines like sipuleucel-T or pTVG-HP might be best deployed earlier in the course of disease. Of note, both sipuleucel-T and pTVG-HP target the same prostate tumor antigen, PAP. It is not clear whether these similar findings are due to targeting of the PAP antigen, or if similar findings would be observed with vaccines targeting other antigens.

Previous trials with anti-tumor vaccines have suggested that they might impact the efficacy of subsequent therapies. In the case of prostate cancer, in one trial of patients with mCRPC treated with the sipuleucel-T vaccine, 51 patients treated with vaccine and 31 patients treated with placebo were followed long-term after treatment. An overall survival benefit was observed for patients that received standard subsequent docetaxel-based chemotherapy compared with patients receiving placebo (HR = 1.9, p = 0.023).^20^ Similarly, in 34 patients with mCRPC treated with the whole tumor-cell vaccine GVAX, 13 patients who went on to receive a taxane-based chemotherapy after receiving vaccine had a median overall survival of 35.2 months vs. 17.2 months for those who did not receive chemotherapy.^21^ In preclinical studies we had found that mice bearing prostate tumors and receiving ADT had greater anti-tumor efficacy if they received an anti-tumor vaccine prior to ADT rather than after, delaying the time to castration-resistant tumor growth.^22^ Findings in the current report are consistent with these prior reports that treatment with pTVG-HP may have impacted the time to castration-resistant disease, potentially by modulating the efficacy of subsequent ADT. While these findings were not statistically significant between the study arms (p = 0.116), partly due to the small sample size, the median time to castration-resistant prostate cancer of 4.7 years observed in patients treated with vaccine was still longer than the expected 2–3 years which was observed in placebo-treated individuals.^23^

The current analysis has several limitations, primarily that this was an unplanned analysis of a previously conducted trial. As such, the evaluation of overall survival was not anticipated, and the trial was not powered to prospectively detect potential differences in survival. In addition, we were unfortunately unable to obtain data from all treatment sites for longer term analysis which may have resulted in selection bias. Another limitation is the heterogeneity in systemic therapies used following castration resistance which may have impacted differences in overall survival. Notwithstanding, the magnitude of the difference in survival observed suggests that this agent or approach should be prospectively evaluated in confirmatory trials, as there was likely single-agent activity that was not originally detected due to the choice of study endpoint. Moreover, the overall survival benefit observed was greater than what was observed in the EMBARK trial,^6^ suggesting that there is still a role for non-hormonal therapies in patients with biochemically recurrent disease. In particular, the use of vaccines prior to ADT and/or enzalutamide might further prolong the time to castration resistance, as has been suggested in murine models and a clinical trial.^22,24^ However, given that standard treatments for this stage of biochemically recurrent prostate cancer have changed over the last several years, a repeat trial with a 15-year analysis to detect differences in overall survival would not be feasible. Our findings that the magnitude of effect may be greater in earlier stages of disease support the concept of evaluating anti-tumor vaccines in the adjuvant setting of prostate cancer, a stage of disease for which there are limited options and time to recurrence may be a more feasible endpoint. We are currently exploring vaccination with or without immune checkpoint blockade in the neoadjuvant setting (NCT04989946). In any case, our trial highlights that immunotherapy agents, and vaccines in particular, likely require different metrics of efficacy. It has been suggested that overall survival is a better endpoint for cancer vaccines than shorter term endpoints such as objective response or time to next therapy.^25^ While this complicates the development of anti-tumor vaccines in early stages of prostate cancer, given the long natural history of the disease, our findings underscore that MFS may not be a surrogate measure of longer term efficacy in biochemically recurrent prostate cancer.

## Supplementary Material

Supplementary Files

This is a list of supplementary files associated with this preprint. Click to download.

- SupplementalInformation.docx

## Figures and Tables

**Table 1:** Demographics. Baseline characteristics for all patients enrolled^7^ and for the subset of patients with long-term follow-up.

	All patients		Long-term follow-up cohort	
	GM-CSF	pTVG-HP (n = 48)	GM-CSF	pTVG-HP (n = 30)
	(n = 49)		(n = 29)	
Age (years)				
Median:	65	65	65	66
Range:	53–82	44–78	53–82	49–78
Median:	71	71	65	66
Range:	56–86	46–82	53–82	49–78
Race / Ethnicity				
Caucasian	46 (94%)	43 (90%)	29 (100%)	28 (93%)
African American	1 (2%)	1 (2%)	0 (0%)	1 (3%)
Asian/Pacific Islander	0 (0%)	1 (2%)	0 (0%)	0 (0%)
American Indian/Alaska Native	0 (0%)	1 (2%)	0 (0%)	1 (3%)
Unknown	2 (4%)	2 (4%)		
Prior treatment				
Prostatectomy	34 (69%)	41 (85%)	18 (62%)	25 (83%)
Radiation therapy				
Primary treatment	20 (41%)	12 (25%)	11 (38%)	5 (17%)
Adjuvant / salvage	28 (57%)	31 (65%)	17 (59%)	22 (73%)
Androgen deprivation	13 (27%)	15 (31%)	9 (31%)	6 (20%)
Chemotherapy	1 (2%)	1 (2%)		
Gleason Score				
≤ 7	34 (69%)	32 (67%)	18 (62%)	22 (73%)
> 7	15 (31%)	16 (33%)	11 (38%)	8 (27%)
Baseline PSA				
2.0–10.0 ng/mL	42 (86%)	40 (83%)	23 (79%)	26 (87%)
> 10 ng/mL	7 (14%)	8 (17%)	6 (21%)	4 (13%)
Baseline PSA DT				
<3 months	10 (20%)	11 (23%)	9 (31%)	7 (23%)
3–6 months	22 (45%)	21 (44%)	9 (31%)	14 (47%)
6–12 months	17 (35%)	16 (33%)	11 (38%)	9 (30%)

## Data Availability

The data generated and/or analyzed during this study are available from the corresponding author on reasonable request.
